# Synthesis and Ultraviolet Visible Spectroscopy Studies of Chitosan Capped Gold Nanoparticles and Their Reactions with Analytes

**DOI:** 10.1155/2014/184604

**Published:** 2014-08-19

**Authors:** Norfazila Mohd Sultan, Mohd Rafie Johan

**Affiliations:** Nanomaterials Engineering Research Group, Advanced Materials Research Laboratory, Department of Mechanical Engineering, University of Malaya, 50603 Lembah Pantai, Kuala Lumpur, Malaysia

## Abstract

Gold nanoparticles (AuNPs) had been synthesized with various molarities and weights of reducing agent, monosodium glutamate (MSG), and stabilizer chitosan, respectively. The significance of chitosan as stabilizer was distinguished through transmission electron microscopy (TEM) images and UV-Vis absorption spectra in which the interparticles distance increases whilst retaining the surface plasmon resonance (SPR) characteristics peak. The most stable AuNPs occurred for composition with the lowest (1 g) weight of chitosan. AuNPs capped with chitosan size stayed small after 1 month aging compared to bare AuNPs. The ability of chitosan capped AuNPs to uptake analyte was studied by employing amorphous carbon nanotubes (*α*-CNT), copper oxide (Cu_2_O), and zinc sulphate (ZnSO_4_) as the target material. The absorption spectra showed dramatic intensity increased and red shifted once the analyte was added to the chitosan capped AuNPs.

## 1. Introduction

Gold nanoparticles (AuNPs) have glowing prospects in many applications due to their distinctive optical, electronic, and electrical properties [1]. AuNPs display intense colours when induced by incident light field. These were contributed by collective electron oscillation that gives intensification to the surface plasmon resonance (SPR) absorption.

There are various techniques to produce AuNPs such as microemulsion, reversed micelles, seeding growth, sonochemistry, photochemistry, radiolysis, and direct chemical reduction [2–4]. The most simple, economical, and powerful synthesis is the direct chemical reduction method. In the case of AuNPs, chemical reduction routes generate zerovalent gold colloids from gold precursors [5].

**Figure 1:**
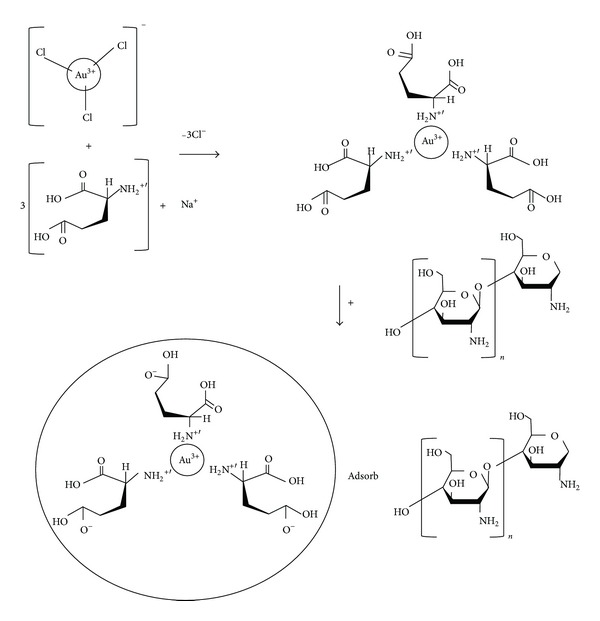
Schematic diagram of gold precursor reduction by MSG and capping the gold particle surfaces with chitosan.

The invention of zerovalent gold colloids was pioneered by Turkevich et al. [6] and later refined by Frens [7] in which the ratio of gold precursors to citrate was varied. Brust-Schiffin [8] commenced the synthesis of AuNPs in organic solvents which involves a phase transfer agent such as toluene. The above conventional methods had many shortcomings which contributed to explorations of other reducing agents and alternative routes. The synthesis of AuNPs through Turkevich et al. approaches takes a longer time (1 hr) for gold salt reduction. While the use of organic solvents in Brust-Schiffin method leaves them inapt for detecting biomolecules and biological surfaces like proteins and saccharides [9], various chemicals had been exploited as reducing agent to produce zerovalent gold colloids such as amino acid derivatives like lysine and valine but without success. However, other acidic amino acid derivatives such as aspartic acid [10] and monosodium glutamate (MSG) [11] are competent in reducing gold salt (Figure 1). Sugunan and Dutta [11] produced AuNPs by emphasizing on lower molar ratio of MSG.

AuNPs have compelling tendency to flocculate due to their van der walls forces. However, the agglomeration can be hindered by introducing a repulsive force between the particles. In this light, the use of stabilizer as a repulsive force came into the picture. The use of chitosan as a stabilizer was reported elsewhere [12]. Chitosan contributed the steric hindrance to stabilize the nanoparticles as shown in Figure 1. The amino group presence in its polycationic structure activates steric hindrance, thus ensuring strong stability over long durations [12]. For most biological applications, chitosan possesses many attractive functional groups such as biotin [13], aptamers [14, 15], concanavalin (con-A) [16], and bovine serum albumin [17, 18]. However, proteins have a downside as they are expensive although they were widely exploited and offer excellent characteristics. Remarkably, chitosan possesses similar ability as proteins and manipulations of its properties have not been fully extended for numerous applications. Chitosan is accessible for cross-linking through its boundless amino group and its cationic features allowing the ionic cross-linking to take place with multivalent elements. The most promising features of chitosan are its solubility in aqueous acidic solutions [19]. The description of chitosan agrees with the aims of the research to manufacture a readily biocompatible and nontoxic chitosan capped gold nanoparticles.

Sugunan et al. [9] had employed chitosan as stabilizer for silver nanoparticles for heavy metal ion sensor, yet the thermodynamically proficiency of chitosan had not been investigated. Moreover, the performance of chitosan adsorption on the surface of AuNPs has not been studied. In this paper, we report the synthesis of AuNPs and its stabilization mechanism using chitosan. By exploiting the chemistry of amine and chitosan, we have shown that the AuNPs can be prepared in water by complexation of high molar ratio glutamic acid molecules with gold precursors stabilized by the adsorption of chitosan on the surface of AuNPs. Preparation of AuNPs capped with chitosan was carried out in a single-pot process and the resulting particles were thoroughly characterized. The stability of chitosan was furthered studied and discussed.

## 2. Experimental Section 

### 2.1. Materials

Gold (III) chloride (AuCl_3_), acetic acid, and monosodium glutamate (MSG) (99% Na salt of L-glutamic acid) were purchased from Acros Organics. Meanwhile, chitosan (industrial grade) was purchased from Easter Holding Co. Ltd. with deacetylation degree of 80%. All chemicals were used without further purifications and all the solutions were prepared with distilled water.

### 2.2. Preparation of AuNPs

2 mL of 5 mM AuCl_3_ solution (0.1517 g in 100 mL water) was stirred and heated to 100°C. Then, 3 mL of 50 mM Na salt of L-glutamic acid solution (MSG) (0.9357 g in 100 mL water) is quickly poured into the gold solution. The solution was stirred continuously until the colour changed from pale red to intense red. The steps were repeated with 100, 150, 200, 250, and 300 mM of MSG. Another set of samples was prepared for observing the aging behaviour of the AuNPs. The samples were left in ambience temperature for a month.

### 2.3. Preparation of Chitosan Capped AuNPs

The chitosan solution was prepared by mixing the said amount of blended chitosan powder as purchased with distilled water and adequate amount of acetic acid. The solution was stirred at room temperature until the chitosan powder had completely dissolved in the water. 990 *μ*L of chitosan solution (1 g of chitosan in mixture of 100 mL water and 150 *μ*L acetic acid) was then added to the as-synthesized 50 mM of MSG reduced AuNPs. A visible change of colour occurred immediately. The heating was discontinued to allow the solution to reach the ambient temperature. The steps were repeated with different concentrations of MSG (100, 150, 200, 250, and 300 mM). Another set of samples was prepared for observing the aging behaviour of the chitosan capped AuNPs. The samples were left in ambience temperature for a month.

### 2.4. Preparation of Amorphous Carbon Nanotubes (*α*-CNTs) Chitosan Capped AuNPs

The synthesis procedure of *α*-CNTs is followed by Tan et al. [20]. The procedure was instigated with mixture of 8 mL of ethyl alcohol (90%), 4.2 g of NaBH_4_ (99.99%), and 15 mL of 1 M NaOH in a 25 mL flask. The solution was further stirred for the next 45 minutes before being transferred to a Parr reactor with capacity of 200 mL. The reactor was heated inside a furnace up to 200°C and held for 2 hours under scaled condition. The Parr reactor was allowed to cool to ambient temperature and the precipitate was washed thoroughly with alcohol and deionised water. The precipitate was then dried in the vacuum oven. *α*-CNTs were added to the optimum condition of chitosan capped AuNPs solution (1 g of chitosan powder and 100 mM of MSG).

### 2.5. Preparation of Copper Oxide-Chitosan Capped AuNPs

0.005, 0.01, 0.05, 0.1, and 0.5 g of purchased copper oxide powder were added to optimum condition of chitosan capped AuNPs solution (1 g of chitosan powder and 100 mM of MSG).

### 2.6. Preparation of Zinc Sulphate-Chitosan Capped AuNPs

0.005, 0.01, 0.05, 0.1, and 0.5 g of purchased zinc sulphate powder were added to optimum condition of chitosan capped AuNPs solution (1 g of chitosan powder and 100 mM of MSG).

### 2.7. Characterizations of AuNPs

Transmission electron microscope (Libra 120 TEM using accelerating voltage of 400 kV) was employed to assess the particles size and distribution of the particles. The optical properties of gold dispersions were investigated by UV-Vis spectrophotometer using UVIKON 923 UV-Vis spectrophotometer.

## 3. Results and Discussion

### 3.1. TEM Analysis

**Figure 2:**
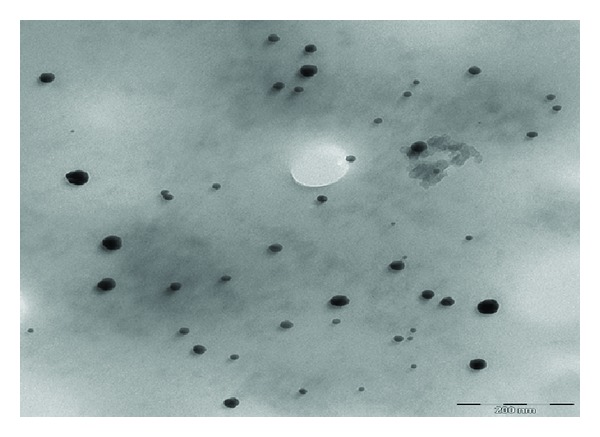
TEM image of AuNPs reduced with 100 mM of MSG.

**Figure 3:**
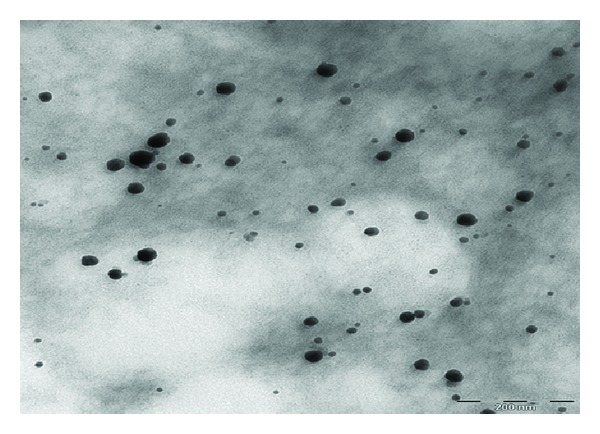
TEM image of AuNPs reduced with 200 mM of MSG.

**Figure 4:**
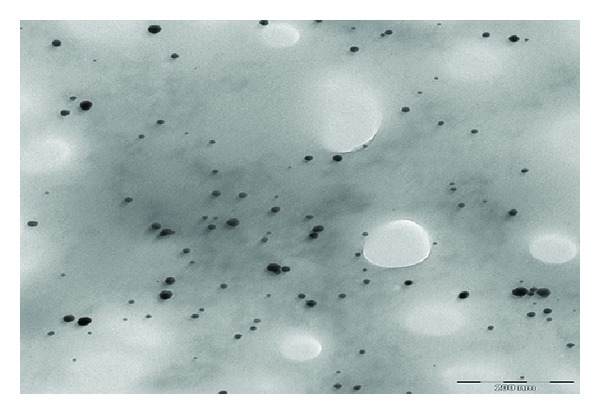
TEM image of AuNPs reduced with 300 mM of MSG.

Figures 2, 3, and 4 show the TEM images for AuNPs prepared at different concentrations of MSG. The particles are nearly spherical with high dispersibility. The average size of particles for 100, 200, and 300 mM MSG is 18, 15, and 9 nm, respectively. It is clearly shown that high molar of MSG produces smaller particle size.

**Figure 5:**
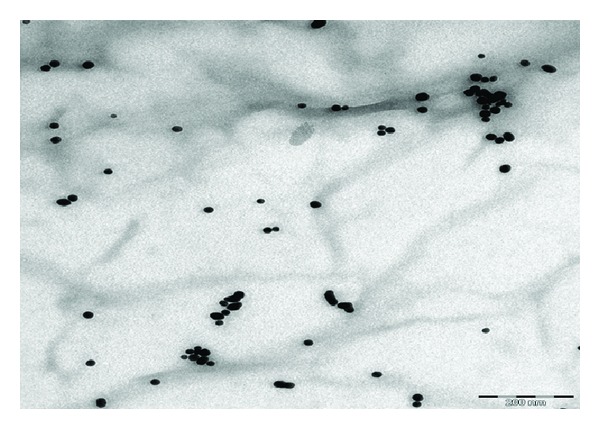
TEM image of chitosan capped AuNPs.

The role of chitosan in steric mechanism has been verified by the TEM image shown in Figure 5. The chitosan which resembles a spider web infused a repelling force between the AuNPs separating them apart unlike the bare AuNPs (Figures 2–4). The average interparticles distance increases to 96 nm due to wrapping of chitosan around the AuNPs.

### 3.2. UV-Vis Spectroscopy Analysis

#### 3.2.1. Effect of Concentration of Reducing Agent MSG

**Figure 6:**
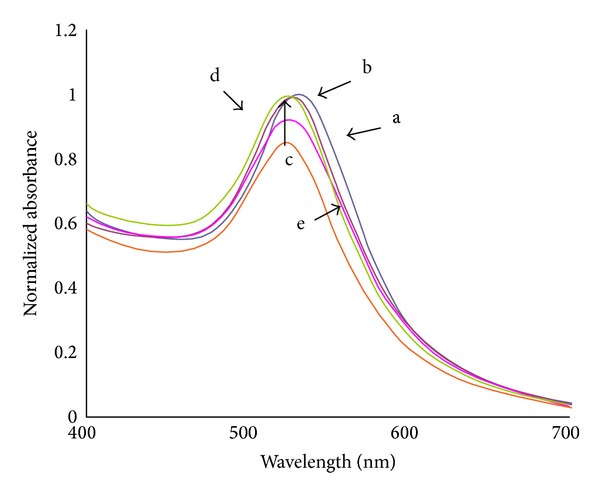
Absorption spectra of AuNPs with various concentrations of reducing agent MSG: (a) 100 mM; (b) 150 mM; (c) 200 mM; (d) 250 mM; (e) 300 mM.

Figure 6 shows the absorption spectra of AuNPs at various concentrations of MSG. The surface plasmon resonance (SPR) peaks are shifted to the smaller wavelengths indicating the reduction in particle sizes. This result is in good agreement with the TEM images in Figures 2–4. The symmetrical shape of the absorption spectra indicates that sample has a narrow particle size distribution.

#### 3.2.2. Effect of Chitosan as Stabilizer

**Figure 7:**
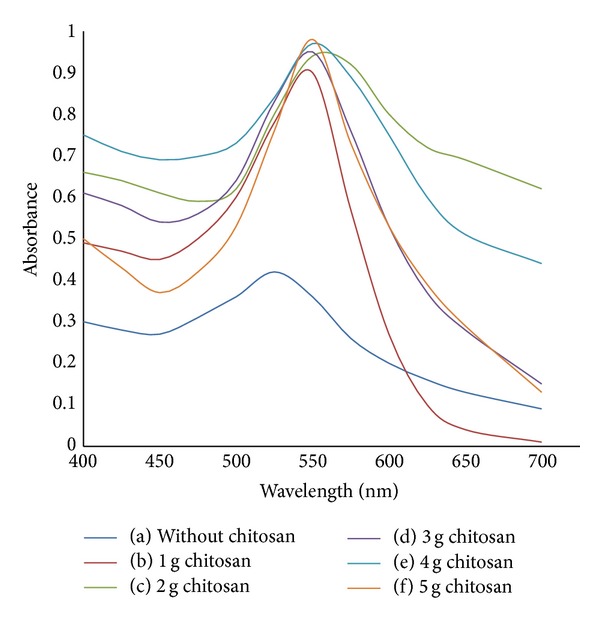
Absorption spectra of bare and increased weight of chitosan capped AuNPs at the optimum concentration of reducing agent MSG: (a) 0 g chitosan; (b) 1 g chitosan; (c) 2 g chitosan; (d) 3 g chitosan; (e) 4 g chitosan; (f) 5 g chitosan.

Figure 7 shows the absorption spectra of bare and increased weight of chitosan capped AuNPs at the optimum concentration of reducing agent MSG (100 mM). The absorbance of chitosan capped AuNPs is higher than bare AuNPs. The SPR peak is shifted to the longer wavelength for chitosan capped AuNPs. This red shifted trend is continued for samples with increasing weight of chitosan. The absorbance for chitosan capped AuNPs is slightly increased with the increase of chitosan weight. The attachment of chitosan on the surface of AuNPs affected their optical properties.

#### 3.2.3. Effect of Aging

**Figure 8:**
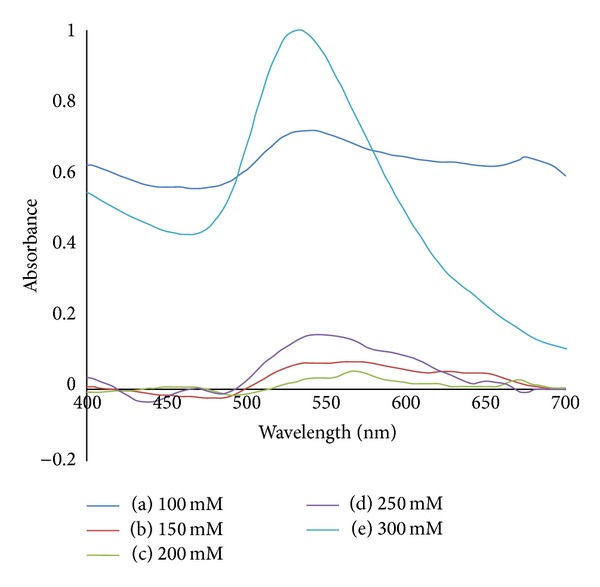
Absorption spectra of AuNPs with various concentrations of reducing agent MSG after 1 month aging time: (a) 100 mM; (b) 150 mM; (c) 200 mM; (d) 250 mM; (e) 300 mM.

Figure 8 shows the absorption spectra of AuNPs with various concentrations of MSG after 1 month aging time. The SPR peaks are shifted to longer wavelength compared to their counterparts in Figure 6. The same goes for their FWHM values which show more broadened peak after 1 month ageing time. This indicates that the AuNPs size and their particle size distribution are increased after aging.

**Figure 9:**
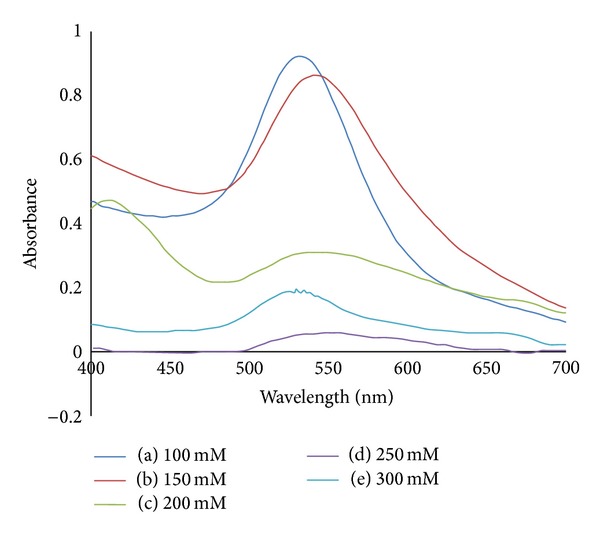
Absorption spectra of chitosan capped AuNPs with various concentrations of reducing agent MSG after 1 month aging time: (a) 100 mM; (b) 150 mM; (c) 200 mM; (d) 250 mM; (e) 300 mM.

Figure 9 shows the absorption spectra of bare and chitosan capped AuNPs for various concentrations of MSG after 1 month ageing time. The SPR peaks are shifted to the smaller wavelength compared to the bare AuNPs (Figure 8). The same goes for their FWHM values which are smaller than the FWHM values of the bare AuNPs. These absorption spectra have highlighted the role of chitosan adsorption on the AuNPs surfaces, in which their particle size stays small even after 1 month aging time. Chitosan preserves stability and hinders agglomeration of AuNPs. The FWHM and other experimental results are listed in Table 1.

#### 3.2.4. Effect of Amorphous Carbon Nanotube (*α*-CNTs)s, Oxides, and Sulphate to Chitosan Capped AuNPs

**Figure 10:**
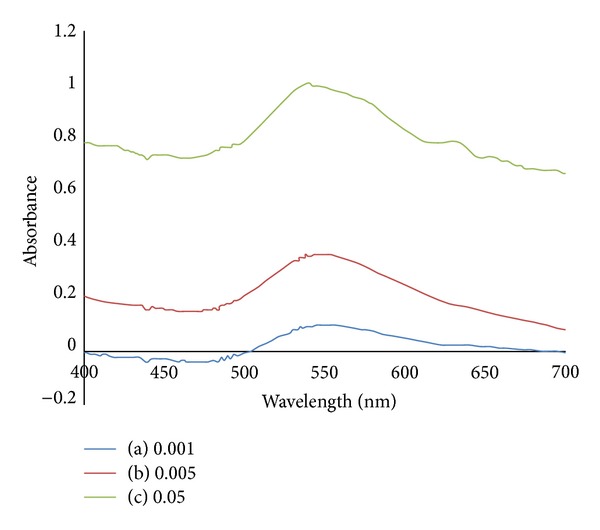
Absorption spectra of chitosan capped AuNPs mixed with different weights of *α*-CNTs: (a) 0.001 g; (b) 0.005 g; (c) 0.05 g.

Figure 10 shows the SPR peaks of chitosan capped AuNPs adjourned at 549 nm for three different weights of *α*-CNTs. The SPR peak intensity rises as the weight of *α*-CNTs increases. This phenomenon can be explained with regard to the fact that AuNPs are very sensitive in the weight change of *α*-CNTs upon exposure. High surface ratios of AuNPs contribute to the sensitivities and make them more reactive and able to uptake the analyte.

**Figure 11:**
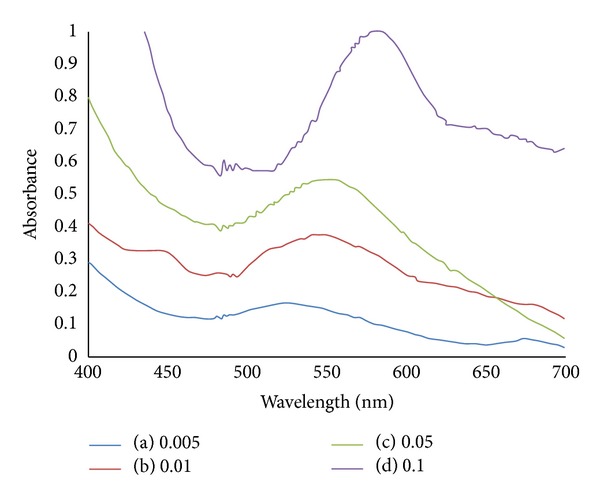
Absorption spectra of chitosan capped AuNPs mixed with different weights of copper oxide: (a) 0.005 g; (b) 0.01 g; (c) 0.05 g; (d) 0.1 g.

Figure 11 shows the absorption spectra of chitosan capped AuNPs mixed with different weights of copper oxide. The SPR peaks are shifted from 521 to 577 nm as the weight of copper oxide increases. This clearly shows the complexation of chitosan capped AuNPs towards the addition of copper oxide. The enlargement of the particles uptake can be underlined as the peak intensity also shows dramatic increase as the weight of copper oxide increases.

**Figure 12:**
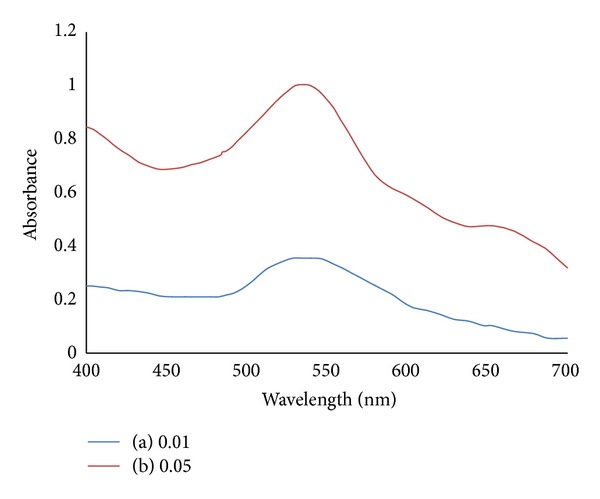
Absorption spectra of chitosan capped AuNPs mixed at different weights of zinc sulphate (a) 0.01 g; (b) 0.05 g.

Figure 12 shows the absorption spectra of chitosan capped AuNPs at different weights of zinc sulphate. The spectra also successfully show analyte particles entrapment by chitosan capped AuNPs. The SPR peaks intensities are increased as the weight of zinc sulphate added to the chitosan capped AuNPs increases.

## 4. Conclusion

We have successfully synthesized chitosan capped AuNPs via chemical reduction technique. We have also revealed that AuNPs revolutionize their dimension and optical behaviour through variation of parameters such as concentration of reducing agent, weight of stabilizer, and aging time. Chitosan concentration plays an important role in imparting extra hindrance strength. The particles stability was contributed by chitosan even after 1 month of aging. The chitosan capped AuNPs were able to uptake analyte such as *α*-CNTs, copper oxide, and zinc sulphate.

## Figures and Tables

**Table 1:** Experimental values of SPR peaks for bare and chitosan capped AuNPs with full width half maximum (FWHM) after 1 month ageing time.

Sample	SPR *λ*_max⁡_ (nm)		FHWM (nm)	
Concentration of MSG (mM)	AuNPs	Chitosan capped AuNPs	AuNPs	Chitosan capped AuNPs
100	532	531	51	60
150	544	537	72	52
200	554	539	52	51
250	544	527	96	46
300	534	526	70	57
